# Aerobic exercise as a therapeutic intervention for post-traumatic stress disorder in women who have experienced intimate partner violence: study protocol for an open-label randomised controlled trial

**DOI:** 10.1186/s13063-026-09796-z

**Published:** 2026-05-29

**Authors:** Charlotte Copas, Jen Makovec Knight, Sarah Griffith, Abigail Astridge, Beatriz Duarte Martins, Stuart J. McDonald, Zhibin Chen, Terence J. O’Brien, Sandy R. Shultz, Georgia F. Symons

**Affiliations:** 1 Department of Neuroscience, School of Translational Medicine, Monash University, Melbourne, VIC Australia; 2 Monash-Epworth Rehabilitation Research Centre, Richmond, VIC Australia; 3 Centre for Trauma and Mental Health Research, Health Sciences and Human Services, Vancouver Island University, Nanaimo, BC Canada; 4 Department of Neurology, Alfred Hospital, Melbourne, VIC Australia; 5 Institute on Aging & Lifelong Health, Faculty of Health, University of Victoria, Victoria, BC Canada

**Keywords:** Exercise, Intimate partner violence, Post-traumatic stress disorder, Domestic and family violence

## Abstract

**Background:**

Women with a history of intimate partner violence (IPV) are at risk for post-traumatic stress disorder (PTSD). The effectiveness of aerobic exercise in reducing PTSD symptom burden has been established in other patient populations; however, its utility in the context of IPV victim-survivors has received little investigation. Therefore, the primary aim of this study is to determine if 4 weeks of daily structured aerobic exercise compared to daily stretching can reduce PTSD symptom burden in women IPV victim-survivors, offering a potential accessible and self-directed treatment avenue.

**Methods:**

The target sample size of 120 women (aged 18–70) with a history of IPV (last instance > 3 months ago) and probable PTSD (via the PTSD Checklist for DSM-5; PCL-5) will be recruited via community advertisement in Melbourne, Australia. The trial will be completed at the Alfred Centre, Monash University. Participants will complete an exercise tolerance test via the Buffalo Concussion Bike Test and then will be randomised into either aerobic exercise or passive stretching. Participants will be instructed to complete 20 min of allocated aerobic exercise or stretching for 4 weeks and given a written diary and a Fitbit smartwatch to track program adherence. The primary outcome, PTSD symptoms via the PCL-5, will be collected at baseline, 1, 2, 3, and 4 weeks (primary endpoint). Secondary outcomes, including additional determinants of health (i.e. sleep, pain, substance use), cognitive testing, concussion-like symptoms in participants with a history of IPV-related brain injury, blood-based biomarkers, and feasibility and adherence to the prescribed program, will also be collected weekly at baseline, 1-, 2-, 3-, and 4-week follow-ups.

**Discussion:**

This trial is an open-label randomised controlled trial to compare the effectiveness of a structured aerobic exercise program to passive stretching in women victim-survivors of IPV with probable PTSD. Results from this trial will help guide the development of individualised, financially accessible, and self-directed care plans for women living with PTSD and other persisting mental and physical health impacts of IPV.

**Trial registration:**

ACTRN12624000893505. Trial registered retrospectively. Registered on 22/07/2024. https://anzctr.org.au/Trial/Registration/TrialReview.aspx?ACTRN=12624000893505

**Supplementary Information:**

The online version contains supplementary material available at 10.1186/s13063-026-09796-z.

## Background

Intimate partner violence (IPV) is a major worldwide healthcare burden that disproportionately affects women. It refers to behaviours by a current or former partner (formal or informal) that cause physical, sexual, or psychological harm and encompasses physical aggression, psychological abuse, and controlling behaviours [1]. Global prevalence of physical and/or sexual violence across a lifetime is 27% [2]. However, meta-analysis suggests that lifetime incidence is significantly increased when also considering psychological IPV, and has been reported up to 37.3% in women aged 16 and over [3].

Persistent mental health conditions are a significant and ongoing challenge for IPV victim-survivors, with post-traumatic stress disorder (PTSD) being one of the most prevalent. PTSD affects an estimated 63.8% of survivors, irrespective of the type of IPV experienced [4]. It is defined as the re-experiencing of a traumatic event in addition to avoidance, negative cognition and mood, alterations in arousal and reactivity affecting functioning for longer than 1 month [5]. PTSD has profound consequences for quality of life, health, and productivity, impacting patients’ families, friends, and employers [6]. Consequently, there is a pressing clinical imperative to identify cost-effective and accessible therapeutics to improve health outcomes among this vulnerable population [7, 8]. This research aims to determine whether aerobic exercise can improve outcomes and quality of life in survivors of IPV with PTSD.

Physical activity has been suggested to improve PTSD symptomology in other populations with high rates of gendered violence [9–11]. Existing research has focused on military populations [12, 13], which disproportionally focuses on male combat-related trauma and may not be generalisable to the PTSD experience in women who have experienced IPV [14, 15]. Where female veterans with PTSD have been the focus, 12 weeks of moderate-intensity aerobic exercise (i.e. brisk walking; 4× per week) was shown to improve PTSD and depressive symptoms using a within-subject design [16]. Interestingly, this population included patients with military sexual assault (50%), military combat-related trauma (27%), and 14% IPV-related PTSD [16]. The beneficial effects of aerobic exercise are believed to occur through mechanisms of enhanced neuroplasticity, reduced inflammation, and regulation of the hypothalamic-pituitary-adrenal axis [9]. Improvements attributed to these mechanisms, such as increases in brain-derived neurotrophic factor (BDNF) protein expression [17] and decreased concentrations of pro-inflammatory cytokines [18, 19], have been demonstrated in rodent models of PTSD and in humans that performed aerobic exercise [17]. However, there is a paucity of research related to the efficacy and mechanisms of aerobic exercise within the context of IPV survivors with PTSD. Therefore, randomised clinical trials are required to strengthen the evidence of aerobic exercise for PTSD among this population [9].

Here, we will investigate the benefit of 4 weeks of structured aerobic exercise interventions on PTSD symptomology, in comparison to control intervention of a program of passive stretching. In addition, we will also assess whether cognition and other biopsychosocial measures are influenced by the intervention. Blood biomarkers related to inflammation and growth factors will be measured to provide mechanistic insights.

### Objectives

The primary objective is to determine the effects of a 4-week program of structured aerobic exercise on PTSD symptom burden in women IPV victim-survivors compared to those who complete a program of 4 weeks of passive stretching. The secondary objective is to determine the effects of the exercise intervention on biopsychosocial measures (i.e. quality of life, depression, anxiety and stress, concussion-like symptoms, sleep), cognitive performance, and blood biomarkers related to inflammation and growth factors compared to passive stretching.

## Methods

### Design

This trial is an open-label randomised controlled superiority trial to compare the effectiveness of aerobic exercise to passive stretching in women victim-survivors of IPV with probable PTSD. Trial design is such that participants will be randomly allocated using simple randomisation (ratio 1:1) in parallel to either aerobic exercise intervention or control passive stretching intervention. This trial will be taking place at the Alfred Centre at Monash University in Melbourne, Australia. No patients or members of the public were involved in the design, conduct, or reporting of this protocol due to the sensitive nature of the study population.

### Population

Women aged 18–70 with a history of IPV (last instance > 3 months ago) and probable PTSD (via the PTSD Checklist for DSM-5; PCL-5) will be recruited.

### Inclusion criteria

1. Women aged between 18 and 70 years
2. Self-report history of IPV, with the last occurrence being at least 3 months prior
3. Probable PTSD as measured by a score of >31 on the post-traumatic stress disorder checklist for DSM-5 (PCL-5)
4. Have daily access to a smartphone and the internet

### Exclusion criteria

1. Subjects who are not willing or able to exercise due to health or personal reasons (e.g. orthopaedic injury, cervical spine injury, diabetes, known heart disease, or musculoskeletal injuries which could make exercise difficult or painful) via the Physical Activity Readiness Questionnaire (PAR-Q)
2. Increased cardiac risk indicated by two or more of the following:
  - Prior diagnosis of, or currently taking medication for cardiovascular (e.g. beta-blockers), metabolic, or pulmonary conditions
  - Family history of myocardial infarction, coronary revascularisation, or sudden death before 55 years
  - Diagnosis of hypertension
  - Diagnosis of hyperlipidaemia
  - Subjects with peripheral circulatory disorders
3. History of prior head injury as defined by:
  - Any head injury within the last 30 days
  - Moderate or severe TBI, defined as a brain injury with an associated Glasgow Coma Scale score of 12 or less
4. Diagnosis of a neurological disorder (e.g. stroke, multiple sclerosis, epilepsy, brain tumour/cancer, encephalitis, dementia, movement disorder, spontaneous nystagmus, or brain surgery)
5. History of drug or alcohol dependency or abuse within a year before screening by self-report
6. Limited English proficiency precluding completion of measures
7. Significant psychiatric history (e.g. psychiatric hospitalisation, schizophrenia, history of legal trouble for violence)
8. IPV history < 3 months
9. No PTSD symptomology at the initial visit (i.e. asymptomatic)
10. Sustaining a head injury during the research period
11. New (<1 month) or non-stable or pharmacological regimen prior to intervention (regimen must be maintained for the duration of intervention)
12. Exercise-tolerant individuals with a current activity level equal to or greater than the proposed intervention as indicated by modified Godin Leisure-Time Exercise

### Study outline

**Fig. 1:**
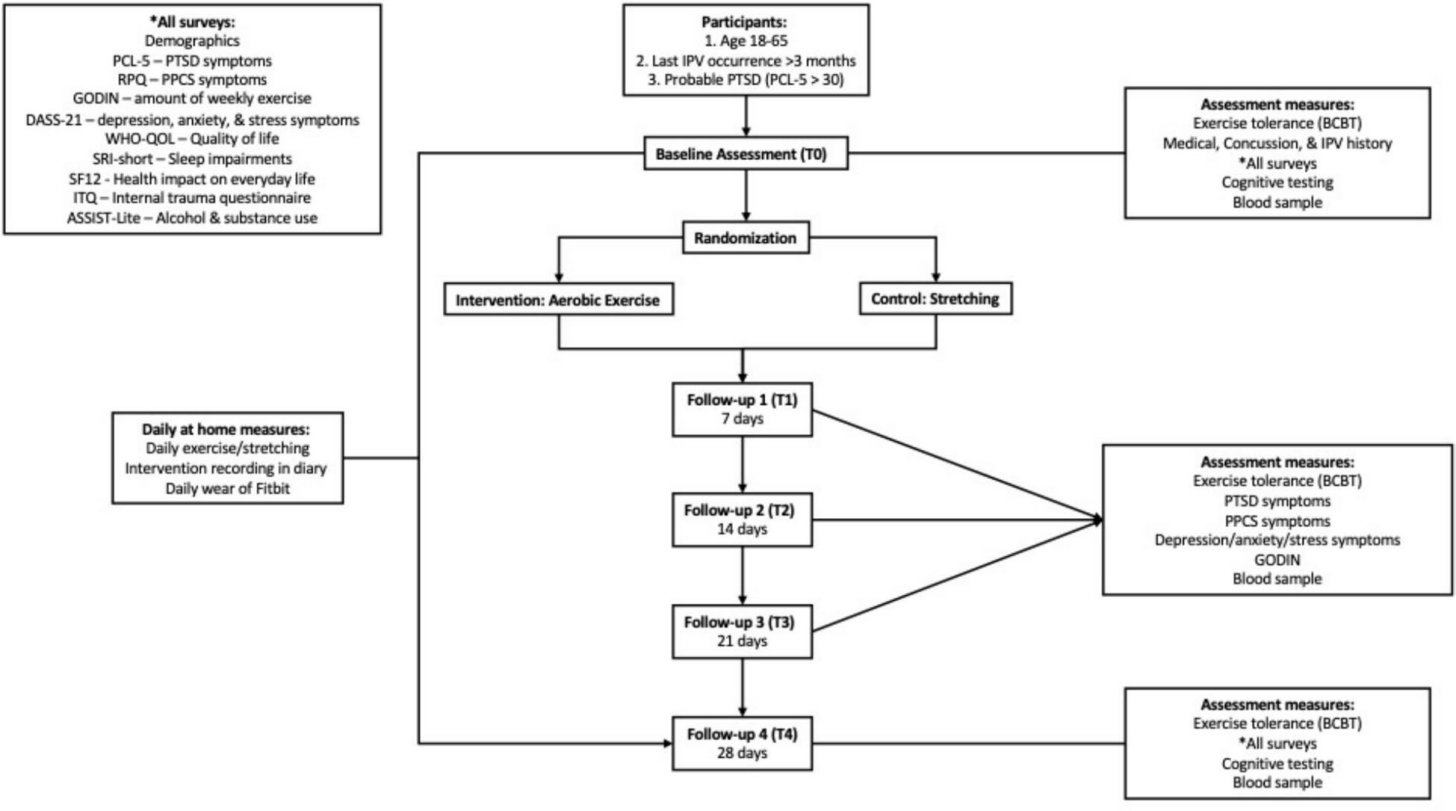
Study timeline flowchart

The trial will encompass a total of 5 sessions over 4 weeks. Participants with low exercise risk will meet a researcher in person at the Alfred Centre for baseline and subsequent weekly follow-up assessments thereafter. Daily exercise or stretching will be performed by the participants at their own discretion. A flowchart of the study timeline is provided in Fig. 1.

### Sample size

Although there are limited studies to base a statistical power calculation, the sample size was determined based on previous sample size estimates from trials using sub-symptom threshold aerobic exercise (STAE) interventions, which ranged from 46 to 96 participants [20, 21]. With an estimated drop-out rate of 20%, we will aim to recruit 120 participants [21].

### Recruitment and screening process

Potential participants will be recruited through advertisement flyers in the community (e.g. hospitals, libraries, sports centres, women’s health and family violence services, community hubs, and rehabilitation clinics), social media, and by contacting participants from ongoing projects. When an individual provides consent to be contacted, a staff member on the research team will conduct a phone call to determine eligibility. During the phone call, participants will be assessed based on the inclusion and exclusion criteria. This will include PTSD symptoms, estimates of physical activity (i.e. Godin Leisure-Time Exercise) [22], cardiac risk, and individual safety in engaging in exercise (i.e. The Physical Activity Readiness Questionnaire; PAR-Q) [23].

### Informed consent

At baseline, participants will be given a verbal summary of the study as well as any potential risks associated with participating. Participants were also given a list of mental health and IPV resources to contact if any aspect of the trial triggers distressing thoughts or feelings. Before commencing baseline surveys, participants will be given written informed consent.

## Interventions

### Aerobic exercise intervention

Participants determined to be exercise tolerant will be instructed to complete 4 weeks [24, 25] of aerobic exercise at the prescribed target heart rate (HR) of 50–65% of age-predicted HR [26, 27]. Participants determined to be exercise intolerant via the Buffalo Concussion Bike Test (BCBT) will be instructed to complete 4 weeks of STAE. The STAE prescription target HR will be 80% of the HR achieved at symptom exacerbation on the BCBT at the most recent visit. All participants will be required to complete 20 min of aerobic exercise of choice at home or in a gym at the individually prescribed HR, monitored by wearing a provided HR tracker (Fitbit Inspire 3). Participants performing STAE will be instructed to stop their exercise session if their symptoms increase by two or more points from their pre-exercise symptom level (on a 10-point Visual Analog Scale) or at 20 min, whichever comes first. All participants will be encouraged to perform 5 min of chosen warm-up and cool down. They will be told to rest apart from the prescribed exercise and not participate in other physically engaging activities. A 4-week intervention was chosen to align with the interventions’ previous interventions using aerobic exercise as a therapeutic intervention for brain injury [24, 25].

$$Max\;Heart\;Rate\;=\;210\;-\;0.79\lbrack Current\;Age\rbrack$$

$$\begin{array}{c}Goal\;heart\;rate\;=\;(Max\;Heart\;Rate\;-\;Resting\;Heart\;Rate)\;x\;Goal\;Percent\;Effort\;+\\Resting\;Heart\;Rate.\end{array}$$

$$\begin{array}{c}\mathrm{Example}:\;45-\mathrm{year}-\mathrm{old}\;\mathrm{woman},\;\mathrm{resting}\;\mathrm{heart}\;\mathrm{rate}\;50\mathrm{bpm},\;\mathrm{goal}\;\mathrm{percent}\;\mathrm{effort}\;60\%\\210\;-\;0.79(45)\;=\;174\\(174-50)\;x\;0.60+50=124+/-10\mathrm{bpm}\end{array}$$

### Placebo stretching intervention

Participants assigned to the control passive stretching group will be instructed to follow a prescribed stretching program. This group will be provided with a booklet containing a gentle, whole-body, progressive stretching program (with pictures and instructions) that does not considerably elevate HR to perform for 20 min per day for 4 weeks. Participants will be asked to return weekly to re-evaluate performance on the BCBT. All participants will be encouraged to perform 5 min of chosen warm-up and cool down. They will be told to rest apart from the prescribed exercise and not participate in other physically engaging activities. Following 4 weeks of passive stretching intervention, participants will be re-evaluated for exercise intolerance and offered the option to participate in the 4-week aerobic exercise intervention.

## Outcomes

### Primary outcome

The primary outcome of this trial is PTSD symptom burden measured by the PTSD Checklist for DMS-5 (PCL-5) (Table 1). The PCL-5 is a validated and reliable survey for measuring severity of PTSD symptoms and has been used to measure symptom burden in IPV victim-survivors in previous studies [28–30]. The PCL-5 is composed of 20 questions to determine how much a participant was bothered by common PTSD symptoms in the past 3 months. Each question/symptom is ranked on a five-point Likert scale from “0”—not at all to “4”—extremely, with the highest possible value being 80. Probable PTSD was described as a PCL-5 score of >31.

### Secondary outcomes

#### Biopsychosocial questionnaires

Secondary biopsychosocial outcome measures of symptoms of depression, anxiety, and stress (Depression, Anxiety, and Stress Scale; DASS-12) [31], sleep impairment (Sleep Related Impairment—short; SRI-short) [32], pain (Brief Pain Inventory—short; BPI-short) [33], overall health (12-item Short Form Survey; SF-12) [34], complex post-traumatic stress disorder (Internal Trauma Questionnaire; ITQ) [35], alcohol and psychoactive substance use (Alcohol, Smoking, and Substance Involvement Screen Test—lite; ASSIST-lite) [36], and quality of life (World Health Organisation Quality of Life—abbreviated; WHOQOL-BREF) [37]. All secondary outcome measures are included in Table 1.

**Table 1:** Primary and secondary outcome measures

Outcome	Outcome measure
Primary	
PTSD symptom burden	The Posttraumatic Stress Disorder Checklist (PCL-5)
Secondary	
Exercise tolerance	Buffalo Concussion Bike Test (BCBT)—Max HR achieved, HRV metrics
Depression, anxiety, and stress	Depression, Anxiety & Stress Symptom Scale (DASS-12)
Complex post-traumatic stress disorder	International Trauma Questionnaire (ITQ)
Substance use	ASSIST-LITE
Pain	Brief Pain Inventory
General health	12-Item Short Form Health Survey (SF-12)
Sleep	Sleep-related inventory—short (SRI-short)
Concussion-like symptom burden	Rivermead Post‑concussion Symptoms Questionnaire (RPQ)
Quality of life	World Health Organisation Quality of Life (WHOQOL-BREF)
Cognitive outcomes	Cognitive performance at baseline compared to follow-up Rey Auditory Verbal Learning Test WAIS-IV Working Memory Index—subtests Digit Span, Letter Number Sequencing Oral Symbol Digit Modalities Task Trail Making Test
Blood biomarkers	Inflammatory factors (e.g. IL-1β, IL-18) Growth factors (e.g. VEGF, BDNF)
Compliance and feasibility	Weekly follow-up survey and verified via Fitbit reports
Safety and tolerability	Adverse events reported throughout 4 weeks during daily exercise

#### Cognitive measures

Cognitive outcome measures include the Test of Premorbid Functioning (TOPF) [38], Rey Auditory Verbal Learning Test (RAVLT) [39], Digit Span (DS) [40], Oral Symbol Digit Modalities Test (O-SDMT) [41], and the Trail Making Test parts A and B (TMT) [42].

#### Concussion-like symptoms

Mild traumatic brain injuries (mTBI) are reported in up to 92% of women IPV survivors [43], with non-fatal strangulation events and subclinical head traumas also commonly described and potentially contributing to brain pathology. Aerobic exercise has proven to promote recovery in adolescents with persisting concussion symptoms due to sports-related concussions [26, 44–46], and modulate concentrations of brain-specific and systemic blood biomarkers associated with inflammation and vascular and neuronal growth [47–50], many of which have been shown to be dysregulated in individuals with PTSD [51–53] and in rat models of IPV-related brain injury [54]. Therefore, here we will also be documenting each participant’s history of brain injury and measuring the impact of the intervention on concussion symptoms (Rivermead Post-Concussion Symptom Questionnaire; RPQ) [55].

#### Blood-based biomarkers

Approximately 30 ml of venous blood will be collected from each participant at baseline and all follow-up assessments by a trained staff member or physician. All blood samples will be centrifuged to collect serum and plasma, coded with the participant’s unique “Study ID”, and stored in a −80-degree freezer until analysis. Access to biomarker data will be restricted to only the investigators of the study. Biomarkers of interest include those associated with inflammation (e.g. IL-1β, IL-18) and growth factors (e.g. BDNF, VEGF).

#### Exercise tolerance and the Buffalo Concussion Bike Test

Exercise intolerance (i.e. the inability to exercise at or near to age-appropriate maximum HR due to exacerbation of concussion-like symptoms) has been reported in adults with mTBIs and/or PPCS [56] but has not been directly studied in women victim-survivors. Exercise tolerance will be measured using the BCBT and is used to determine the HR at which concussion-like symptom exacerbation occurs (i.e. HR threshold [57, 58]). If a participant is experiencing no symptoms or no exacerbation occurs during the BCBT, the patient is determined to be exercise tolerant. During the BCBT, participants are asked to cycle on a stationary seated bike at a determined revolutions per minute (RPM). Every 2 min, bike resistance will increase, and participants will indicate their rate of perceived exertion using the Borg Ratings of Perceived Exertion (RPE) [59] 6–20 (lowest to highest), and concussion-like symptom onset using the VAS [60]. The height and weight of each individual participant determine bike resistance levels and RPM. HR will be recorded with the Fitbit (Inspire 3) and an electrocardiogram (ECG) (PowerLab 16/36 with Dual Bio Amp; LabChart 8). According to Haider et al. [57] and Leddy and Willer [58], the BCBT is terminated when (1) the participant self-reports reaching an 18 on the RPE, (2) HR on the ECG reaches 80% of age estimated HR-max ((220 − age) * 0.8), (3) the participant self-reports a 3-point increase on the VAS, or (4) the exercise test reaches 30 min. Once the test is finished, bike resistance is brought to zero, and the participant will continue to cycle at their own pace for a 2-min cool down. Following the BCBT, participants will be randomised to either the exercise or the stretching intervention using simple randomisation.

## Data collection, management, and analysis

### Trial allocation

This study is an open-label study; therefore, the investigator, site staff, and subjects will not be blinded to treatment allocation. A computer-generated randomisation schedule will be prepared by a statistician prior to the start of the study. Treatment allocation will be made per the randomisation list. After signing the informed consent form, subjects will be allocated a unique subject ID and administered the screening instruments via Research Electronic Data Capture (REDCap). A unique subject ID will be used to identify the subject throughout the study period and on all study-related documentation. After eligibility has been established, subjects will complete all baseline assessments followed by a simple randomisation in a 1:1 ratio to either the aerobic exercise or stretching group. Randomisation will be allocated sequentially based on the pre-determined randomisation schedule and according to their chronological order of inclusion in the study.

### Assessment and collection of outcomes

Participants will be assessed at baseline (day 0) and at the primary endpoint (day 28). During the trial, participants will return and be re-evaluated weekly on PTSD symptoms, BCBT, biopsychosocial questionnaires, and fluid biomarkers. In addition to exercise compliance, medications, and therapies, as well as any adverse events, compliance and feasibility will be evaluated at the weekly follow-up appointment. Cognitive assessment will only be repeated on the final evaluation. All data collection will occur at the Alfred Centre, Monash University. The full study schedule is included (Table 2).

**Table 2:** Study schedule

Study assessments	Screening	Baseline	Intervention period	Weekly follow-up	Final visit
		Day 0	Day 1–6; day 8–13; day 15–20; day 22–27	Day 7, 14, 21	Day 28
Screening					
Eligibility questionnaire	X				
Enrolment					
Inclusion/exclusion criteria review	X	X			
Informed consent		X			
Allocate unique subject study identification		X			
Questionnaires					
Demographic data		X			
Medical history		X			
Ohio State University TBI Identification Method		X			
Godin Leisure-Time Exercise questionnaire		X		X	X
Medications		X		X	X
Current therapies		X		X	X
Exercise tolerance test					
Buffalo Concussion Bike Test (BCBT)		X		X	X
ECG recording		X		X	X
Biopsychosocial					
PTSD symptomology: PCL-5	X	X		X	X
Rivermead Post-Concussion Symptoms Questionnaire (RPQ)		X		X	X
Depression, Anxiety & Stress Symptom Scale (DASS-12)		X		X	X
The Alcohol, Smoking and Substance Involvement Screening Test (ASSIST-LITE)		X			X
Brief Pain Inventory (BPI)		X			X
12-Item Short Form Health Survey (SF-12)		X			X
World Health Organisation Quality of Life (WHOQOL-BREF)		X			X
Sleep-related inventory (SRI)		X			X
International Trauma Questionnaire (ITQ)		X			X
Neuropsychological assessment					
TOPF		X			
RAVLT		X			X
Digit Span		X			X
O-SDMT		X			X
TMT		X			X
Biomarkers					
Blood sample collection		X		X	X
Therapeutic intervention					
Dispense intervention		X		X	
Fitbit—dispense and instructions		X			
Record daily intervention sessions			X		
Return of Fitbit					X
Safety and feasibility assessments					
Adverse event monitoring		X		X	X
Feasibility					X

### Compliance to the intervention

Participants will be withdrawn from the study if there is a change in medications or if they are unable to attend follow-ups. Self-reported compliance to the intervention will be tracked at weekly follow-up visits via REDCap survey. Fitbit data can also verify adherence to exercise protocol and will be quantified via the following formula [61]:

$$\mathrm{A}\mathrm{d}\mathrm{h}\mathrm{e}\mathrm{r}\mathrm{a}\mathrm{n}\mathrm{c}\mathrm{e}=\frac{{(\mathrm{v}\mathrm{o}\mathrm{l}\mathrm{u}\mathrm{m}\mathrm{e})}_{\mathrm{a}\mathrm{c}\mathrm{t}\mathrm{u}\mathrm{a}\mathrm{l}}}{{(\mathrm{v}\mathrm{o}\mathrm{l}\mathrm{u}\mathrm{m}\mathrm{e})}_{\mathrm{p}\mathrm{r}\mathrm{e}\mathrm{s}\mathrm{c}\mathrm{r}\mathrm{i}\mathrm{b}\mathrm{e}\mathrm{d}}}\times 100$$

$$=\frac{{(\mathrm{H}\mathrm{R}\times \mathrm{t}\mathrm{i}\mathrm{m}\mathrm{e}\times \mathrm{f}\mathrm{r}\mathrm{e}\mathrm{q}\mathrm{u}\mathrm{e}\mathrm{n}\mathrm{c}\mathrm{y})}_{\mathrm{a}\mathrm{c}\mathrm{t}\mathrm{u}\mathrm{a}\mathrm{l}}}{{(\mathrm{T}\mathrm{a}\mathrm{r}\mathrm{g}\mathrm{e}\mathrm{t}\mathrm{H}\mathrm{R}\times 20\times 1)}_{\mathrm{p}\mathrm{r}\mathrm{e}\mathrm{s}\mathrm{c}\mathrm{r}\mathrm{i}\mathrm{b}\mathrm{e}\mathrm{d}}}\times 100$$

The numerator represents the volume of exercise performed divided by the volume of exercise prescribed to the participant. Volume actual is determined by multiplying weekly mean HR recorded during home exercise by weekly mean exercise time in minutes by the number of days per week out of 6 completed (i.e. 3 out of 6 days completed = 3/6 = 0.5). Volume prescribed was determined by multiplying weekly target HR by 20 min by days prescribed (every day excluding scheduled visit = 6/6 = 1). Participants who had an adherence rate of 66.67% or more were adherent, whereas participants with a rate lower than 66.67% were considered to be nonadherent [61].

### Attrition and adherence

To ensure attrition and program adherence, participants’ HR will be collected using a Fitbit Versa 3 and monitored using Fitabase (Small Steps Labs, LLC) [62], a software that synchronises and displays Fitbit data in real-time. If it is observed that a participant’s HR does not reach within the prescribed range, a member of the research team will check in via text or email. Participants will also be required to complete a written diary given at each follow-up to document the type of exercise they completed, how long they exercised for, and if any PPCS symptoms were triggered by engaging in exercise. Additionally, participants will be offered a $50 e-gift voucher at baseline and at each follow-up to encourage attendance. Participants will be withdrawn if they are unable to complete daily exercise or attend follow-ups.

### Data management and storage

All data will also be coded in a re-identifiable manner and securely stored via REDCap on the Monash University server. A master code linking re-identifiable details will be kept in a secure password-protected database REDCap. The manager of the linking data is PI Professor Sandy Shultz. Only researchers who are actively contributing to data collection, statistical analysis, and publication will have access to the data. Any hardcopy data will be stored in a locked drawer in the Alfred Centre, accessible only by the investigators. All fluid-based samples will be coded (i.e. re-identifiable) and stored in an ultralow temperature (−80 °C) freezer housed in labs within the Department of Neuroscience, Monash University, until they are analysed. The freezer will only be accessed by the investigators of this study. During the study, participants will be required to wear a Fitbit (Inspire 3) activity tracker. The device must be paired to their mobile phone and synced to the research platform Fitabase. Only data related to HR, steps taken, activity intensity, calories burned, and sleep rating will be collected. No other data from their mobile phone or daily life will be exported to the institute. It is important to note that the collected data will be used solely for the purposes of the study and will be kept confidential. Given that this study involves self-directed aerobic exercise among participants screened via the PAR-Q and assessed for cardiovascular risk, a data safety monitoring board was not considered necessary. Further, as this trial will be conducted at a single site, oversight of the trial will be managed by the research team. As such, a steering committee and data quality committee were not deemed necessary.

Any amendments to the study protocol will be sent to the Monash Human Research Ethics Committee and will not be implemented until approval. Amendments will also be sent to the PI to be added to the Investigator Site File. Any deviations from the approved protocol will be documented using a breach report form.

### Adverse event reporting

Participants will have the opportunity to record any adverse events that occurred during trial at each weekly follow-up appointment. Any severe adverse events are recorded on a form on REDCap and reported to Monash Human Ethics. Non-serious events will be recorded on a REDCap form and in patient notes.

### Data analysis

Primary and secondary outcomes will be assessed between groups at the primary endpoint (week 4) using two-sample *t*-tests (if normally distributed) or Mann-Whitney *U* tests (if not normally distributed). In addition, variables with multiple time points will also be assessed using generalised linear mixed-effects models. Models will be adjusted for confounders such as age, sex, baseline depression/anxiety/stress, history of migraine or other comorbidities, and cardiovascular fitness (Godin assessment). Feasibility (recruitment, retention, and completion rates), adherence (proportion), and safety (number and types of adverse events) will also be assessed. A statistician will be consulted in the events of missing data, any additional analysis, and to advise on interim analyses and stopping guidelines. Participants who do not complete the full 4-week intervention (i.e. lost to follow-ups) will be excluded from the primary endpoint analysis however will be retained in the descriptive analyses of the feasibility outcomes. Only the principal investigator will have the authority to terminate the trial.

### Ethics and dissemination

This study has been approved by the Monash University Human Research Ethics Committee (#40295) and registered with the Australian New Zealand Clinical Trials Registry (ID: ACTRN12624000893505). Results from this trial will be disseminated in published articles in peer-reviewed journals as well as in presentations at local and international conferences. Participants will be able to access all published, de-identified data in journal publications. A lay summary of overall study findings, along with the abstract of any publications, can be communicated if they wish via email or post.

## Discussion

Women with a history of IPV are at a high risk of PTSD. While there are trials investigating the effectiveness of yoga and psychomotor therapy in reducing PTSD symptom burden [63–65], to our knowledge there are no clinical trials exploring the effectiveness of aerobic exercise in patient cohorts specific to women who have experienced IPV. The primary aim of this study is to explore the effectiveness of a 4-week aerobic exercise intervention compared to a stretching intervention in relieving PTSD symptom burden in women with a history of IPV. Moreover, this research will determine the impact of aerobic exercise on other common comorbid mental health conditions (i.e. depression and anxiety) as well as cognitive functioning post-intervention. Lastly, weekly blood collections will help uncover potential objective biomarkers for PTSD and evaluate physiological responses to exercise in this population. Results of this trial will inform future approaches in constructing individualised, financially accessible, and self-directed treatment plans for women experiencing long-term mental and physical health challenges due to IPV.

### Trial status

The current protocol is v5, dated 12/05/2025. Recruitment for this study began on 16/05/2024. At the time of submission, we are currently recruiting and enrolling participants in the study. Data collection is expected to be completed on 16/05/2027.

## Supplementary Information

Supplementary Material 1.

Supplementary Material 2.

## Data Availability

Not applicable.

## Abbreviations

ASSIST-lite: Alcohol, Smoking, and Substance Involvement Screen Test—lite
BCBT: Buffalo Concussion Bike Test
BDNF: Brain-derived neurotrophic factor
BPI: Brief Pain Inventory
DASS-21: Depression, Anxiety, and Stress Scale—21 items
DS: Digit Span
ECG: Electrocardiogram
HR: Heart rate
IL: Interleukin
IPV: Intimate partner violence
ITQ: Internal Trauma Questionnaire
O-SDMT: Oral Symbol Digit Modalities Test
PAR-Q: Physical Activity Readiness Questionnaire
PCL-5: Post-Traumatic Stress Disorder Checklist for DSM-5
PPCS: Persisting post-concussion symptoms
PTSD: Post-traumatic stress disorder
RAVLT: Rey Auditory Verbal Learning Test
REDCap: Research Electronic Data Capture
RPE: Borg Ratings of Perceived Exertion
RPM: Revolutions per minute
RPQ: Rivermead Post-Concussion Symptom Questionnaire
SF-12: 12-Item Short Form Survey
SRI: Sleep Related Impairment
STAE: Sub-symptom threshold aerobic exercise
TMT: Trail Making Test
TOPF: Test of Premorbid Functioning
VEGF: Vascular endothelial growth factor
WHOQOL-BREF: World Health Organisation Quality of Life—abbreviated
