# Supplementary material for: Aerobic exercise as a therapeutic intervention for post-traumatic stress disorder in women who have experienced intimate partner violence: study protocol for an open-label randomised controlled trial
Source: Trials. 2026 May 29;27:513. doi: 10.1186/s13063-026-09796-z (PMC13410850; doi:10.1186/s13063-026-09796-z)
Supplement: Supplementary file 1 — Supplementary Material 1. [file 13063_2026_9796_MOESM1_ESM.pdf]

Monash University Human Research Ethics Committee

Approval Certificate

This is to certify that the project below was considered by the Monash University Human Research Ethics Committee. The Committee was satisfied that the proposal meets the requirements of the *National Statement on Ethical Conduct in Human Research* and has granted approval.

**Project ID:**	40295
**Application Type:**	HREC Review
**Project Title:**	Therapeutic intervention for intimate partner violence
**Chief Investigator:**	Dr Georgia Fuller Symons
**Approval Date:**	14/11/2023
**Expiry Date:**	14/11/2028

Terms of approval - failure to comply with the terms below is in breach of your approval and the *Australian Code for the Responsible Conduct of Research.*

1. The Chief Investigator is responsible for ensuring that permission letters are obtained, if relevant, before any data collection can occur at the specified organisation.
2. Approval is only valid whilst you hold a position at Monash University.
3. It is the responsibility of the Chief Investigator to ensure that all investigators are aware of the terms of approval and to ensure the project is conducted as approved by MUHREC.
4. You should notify MUHREC immediately of any serious or unexpected adverse effects on participants or unforeseen events affecting the ethical acceptability of the project.
5. The Explanatory Statement must be on Monash letterhead and the Monash University complaints clause must include your project number.
6. Amendments to approved projects including changes to personnel must not commence without written approval from MUHREC.
7. Annual Report - continued approval of this project is dependent on the submission of an Annual Report.
8. Final Report - should be provided at the conclusion of the project. MUHREC should be notified if the project is discontinued before the expected completion date.
9. Monitoring - the project may be subject to an audit or any other form of monitoring by MUHREC at any time.
10. Retention and storage of data - The Chief Investigator is responsible for the storage and retention of the original data pertaining to the project for a minimum period of five years.

Kind Regards,

Professor Nip Thomson

Chair, MUHREC

CC: Miss Beatrice Duarte Martins, Professor Sandy Shultz, Professor Terence O'Brien, Miss Sarah Griffith, Dr Jen Makovec Knight

List of approved documents:

Document Type	File Name	Date	Version
Supporting Documentation	2023-10-11_IPV Exercise_Ad	11/10/2023	1
Consent Form	Chronic_Exercise_ConsentForm_v1-Oct-2023	17/10/2023	1
Questionnaires / Surveys	Questionnaire	17/10/2023	1
Psychological inventories	2.PCL5	17/10/2023	1
Psychological inventories	3.RPQ	17/10/2023	1
Psychological inventories	5.DASS21	17/10/2023	1
Psychological inventories	6. ASSIST Lite	17/10/2023	1
Psychological inventories	7.BPI	17/10/2023	1
Psychological inventories	8.SF12	17/10/2023	1
Psychological inventories	9.Q-LES-Q-SF	17/10/2023	1
Psychological inventories	10.QOLIBRI_OS	17/10/2023	1

Document Type	File Name	Date	Version
Psychological inventories	11.SRI_PROMIS-SF	17/10/2023	1
Psychological inventories	12.ITQ	17/10/2023	1
Psychological inventories	13.HIT-6-test-english	17/10/2023	1
Psychological inventories	14.PHQ-9	17/10/2023	1
Psychological inventories	15.GAD-7	17/10/2023	1
Psychological inventories	16.FSS	17/10/2023	1
Psychological inventories	17. Dizziness Hanicap Inventory	17/10/2023	1
Psychological inventories	1.TOPF	17/10/2023	1
Psychological inventories	2.RAVLT	17/10/2023	1
Psychological inventories	3.DigitSpan	17/10/2023	1
Psychological inventories	4.SDMT	17/10/2023	1
Psychological inventories	5.TMT	17/10/2023	1
Explanatory Statement	2023-10-19_ExplanatoryStatement_IPV Exercise	17/10/2023	1
Supporting Documentation	2023-10-17_Protocol_Exercise for IPV	17/10/2023	1
Explanatory Statement	2023-11-03_ExplanatoryStatement_IPV Exercise	03/11/2023	2
Supporting Documentation	Protocol_Exercise for IPV - v2_2023-11-03_	03/11/2023	2
Supporting Documentation	Ethics comments replies 3Nov-G	03/11/2023	1
